# Genetic variation in the farnesoid X-receptor predicts Crohn’s disease severity in female patients

**DOI:** 10.1038/s41598-020-68686-9

**Published:** 2020-07-16

**Authors:** Aze Wilson, Qian Wang, Ahmed A. Almousa, Laura E. Jansen, Yun-hee Choi, Ute I. Schwarz, Richard B. Kim

**Affiliations:** 1 Clinical Pharmacology, Department of Medicine, Western University, 339 Windermere Rd, London, ON N6A 5A5 Canada; 2 Gastroenterology, Department of Medicine, Western University, 339 Windermere Road, London, ON N6A 5A5 Canada; 3 Department of Physiology and Pharmacology, Western University, Medical Sciences Building, Rm 216, London, ON N6A 5C1 Canada; 4 Schulich School of Medicine and Dentistry, Western University, 1151 Richmond St, London, ON N6A 5C1 Canada; 5 Department of Epidemiology and Biostatistics, Western University, Kresge Building, Rm K201, London, ON N6A 5C1 Canada

**Keywords:** Genetics, Microbiology, Biomarkers, Inflammatory bowel disease

## Abstract

The farnesoid X receptor (FXR) is implicated in Crohn's disease (CD) pathogenesis. It is unclear how genetic variation in *FXR* impacts CD severity versus genetic variation in nuclear receptors such as pregnane X receptor (PXR) and the multi-drug resistance protein 1 (MDR1, ABCB1). To evaluate *FXR-1G* > *T* as a genomic biomarker of severity in CD and propose a plausible molecular mechanism. A retrospective study (n = 542) was conducted in a Canadian cohort of CD patients. Genotypic analysis (*FXR-1G* > *T*, *MDR1 3435C* > *T* and *PXR -25385C* > *T*) as well as determination of the FXR downstream product, fibroblast growth factor (FGF) 19 was performed. Primary outcomes included risk and time to first CD-related surgery. The effect of estrogen on wild type and variant FXR activity was assessed in HepG2 cells. The *FXR-1GT* genotype was associated with the risk of (odds ratio, OR = 3.34, 95% CI = 1.58–7.05, *p* = 0.002) and earlier progression to surgery (hazard ratio, HR = 3.00, 95% CI = 1.86–4.83, *p* < 0.0001) in CD. Female carriers of the *FXR-1GT* genotype had the greatest risk of surgery (OR = 14.87 95% CI = 4.22–52.38, *p* < 0.0001) and early progression to surgery (HR = 6.28, 95% CI = 3.62–10.90, *p* < 0.0001). Women carriers of *FXR-1GT* polymorphism had a three-fold lower FGF19 plasma concentration versus women with *FXR-1GG* genotype (*p* < 0.0001). In HepG2 cells cotransfected with estrogen receptor (ER) and FXR, presence of estradiol further attenuated variant FXR activity. MDR1 and PXR genotypes were not associated with surgical risk. Unlike *MDR1* and *PXR*, *FXR-1GT* genetic variation is associated with earlier and more frequent surgery in women with CD. This may be through ER-mediated attenuation of FXR activation.

## Introduction

Crohn's disease (CD) is an immune-mediated inflammatory bowel disease defined by remitting and relapsing episodes of intestinal inflammation^1^. CD pathogenesis is complex. Dysregulation of the host immune response in the setting of specific environmental and genetic factors are hypothesized to serve as disease precursors^1,2^. A key component of this inappropriate immune response is the disruption of the mucus layer, the loss of epithelial tight junctions and an increased intestinal permeability and thereby an increase in the intestinal immune system's exposure to bacteria, resulting in an immune response via its innate and adaptive arms^1^. Furthermore, there is a shift in production from anti-inflammatory proteins to pro-inflammatory cytokines through the activation of nuclear transcription factors such as nuclear factor κB (NFκB)^3^. Early in its course, CD is remitting and relapsing, with periods of activity punctuated by debilitating symptoms of abdominal pain, diarrhea, and weight loss, accompanied by biochemical and endoscopic findings of inflammation. As the disease progresses over time, permanent damage to the intestinal structure may result, leading to an irreversible impairment of intestinal function, significant morbidity and long-term disability. Disease severity is marked by the need for and time to surgery, failure of multiple medical therapies, need for hospitalization and the presence of complications such as fistulae or strictures^4^.

Many studies have attempted to link genetic variation in key genes associated with inflammation, xenobiotic metabolism and transport as well as gene regulators of such pathways to CD susceptibility. Several single nucleotide variations (SNVs) in the multidrug resistance-1 (*MDR1*) gene, the pregnane X receptor (*PXR*, also *NR1I2*) gene, and to a lesser extent, the farnesoid X receptor (*FXR*, also *NR1H4*) gene have been evaluated, and some linked to IBD susceptibility with varying degrees of success^5–13^; however, none have emerged as a clinically meaningful marker of IBD presence, drug response or disease severity.

There is now an increasing appreciation of the bile acid-sensing nuclear receptor, FXR, as the master regulator of bile acid homeostasis and transport pathways, intestinal inflammation, intestinal permeability and response to bacterial overgrowth^3,14,15^. Animal models confirm that in the absence of FXR activation, there is an expansion of the bile acid pool and a more severe presentation of chemically-induced colitis, including increased intestinal cellular infiltrate, collagen deposition and expression of inflammatory genes^3,14^. FXR activation modulates fibroblast growth factor (FGF) 19 expression, and plasma FGF19 concentrations have been used as a surrogate marker of FXR activity^16,17^. Moreover, FXR activation in these same models improves intestinal permeability and attenuates the production of pro-inflammatory cytokines such as IL-1β and TNFα via the NFκB pathway^3,14^.

Our group was the first to identify and demonstrate that *FXR-1G* > *T*, a SNV adjacent to the ATG start codon located within the Kozak consensus motif of the *FXR* gene, is linked to reduced transactivation of *FXR* gene targets^18^. Since the Kozak consensus motif ensures ribosomal binding to mRNA transcripts and efficient protein translation, genetic variation in this conserved sequence is associated with decreased protein translation^19^. Previously, Van Mil et al.^20^ demonstrated that the *FXR1-1G* > *T* is associated with reduced FXR protein expression as well as decreased activation of its down-stream targets, citing translational inefficiency as the underlying cause. Accordingly, *FXR-1G* > *T* may have important functional consequences. Interestingly, complete FXR deficiency, while rare, does exist in the human population, and has been shown to result in progressive familial intrahepatic cholestasis (PFIC), associated with coagulopathy, jaundice and a rapid progression to liver failure^21,22^. Such data suggest major loss of function mutations in *FXR* as contributors to CD are unlikely; however, partial loss of function or expression of *FXR* may contribute to CD progression or severity. To date, the role of the *FXR-1G* > *T* SNV has only been evaluated in a CD population in a very limited capacity^11^.

Accordingly, we hypothesized that changes in the intestinal barrier as a result of reduced FXR expression among those who harbor the *FXR-1*T allele are more likely to exhibit a severe CD phenotype compared to G (reference) allele carriers, and thereby experience a more rapid progression to surgery. Alterations in FXR activity may in part be secondary to genetic variation in the *FXR* gene. The aim of this study was to evaluate the utility of *FXR-1G* > *T* as a genomic biomarker of severity in CD. Herein, we demonstrate the clinical impact of the *FXR-1G* > *T* SNV in a large CD population, and provide insights regarding underlying mechanisms.

## Methods

### Study design and participants

This study was a retrospective, single centre, cohort study carried out in 542 patients with CD, who were seen as part of the Personalized Medicine Program at Western University, London, Canada between March 2013 and November 2018 (Flow chart in Supplementary Figure 1). Additionally, all subjects were screened for *MDR1 3435C* > *T* and *PXR -25385C* > *T*. Subjects who underwent a CD-related intestinal resection were also assessed for FGF19 plasma concentration. All eligible subjects were more than 18 years of age, and had a histopathological diagnosis of CD. Patients were excluded from the primary analysis if information pertaining to their medical history was unavailable or unknown or if they had a diagnosis of ulcerative colitis, UC. Other exclusion criteria included a known history of liver or biliary disease, use of cholestyramine or antibiotics within 4 weeks of blood sampling. Subjects excluded from the primary analysis based on a diagnosis of UC were evaluated for surgical risk and progression to surgery based on *FXR-1G* > *T* genotype and are described in the Supplemental Section.

### Demographic and covariate data abstraction

Data collected on subjects from both cohorts included age, sex, weight, smoking history, medical history, duration of disease as well as CD medication exposures (defined as the receipt of, at a minimum, one dose of a CD-related drug as reported by the patient to their treating gastroenterologist) and responses (adverse drug reactions (ADRs), induction of remission, resistance or loss of response). Data relevant to their CD diagnosis was also collected including disease phenotype, disease activity (based on the clinical scoring index, Harvey-Bradshaw Index, HBI) at the time of blood collection, hospitalizations, and history of and time to surgical resection. This information was collected from patient hospital records between the date of diagnosis and the study end period (June 1, 2019).

### Genotypic analysis

Each subject provided a 5 mL venous blood sample used to extract DNA by a standard protocol (MagNA Pure Compact System, Pleasanton, California). Allelic discrimination using TaqMan assays and a 7500 RT-PCR System (Applied Biosystems, Carlsbad, CA) was performed to determine the following genotypes in CD subjects with available DNA (n = 542): *FXR -1G* > *T* (rs56163822; Taqman assay ID C_25598386_10, Thermofisher), *MDR1 3435C* > *T* (rs1045642; Taqman assay ID C_7586657_20, Applied Biosystems), and *PXR* (*NR1I2*) -*25385C* > *T* (rs3814055; Taqman assay ID C_27504984_30, Thermofisher). Genotyping experiments included three positive controls with previously established genotypes and one negative control. For quality control, 5% of samples were genotyped in duplicate. Congruency was seen amongst all duplicated genotypes.

### Fibroblast growth factor 19 quantification

Blood samples were drawn from study subjects and plasma was extracted by centrifugation. All subjects provided blood samples post-operatively. A commercial enzyme-linked immunosorbent assay (ELISA) kit (FGF19 Quantikine ELISA kit, R&D Systems, Minneapolis, MN, US) was used for the colorimetric detection and estimation of FGF19 plasma concentrations following the manufacturer's instructions for subjects with available plasma samples who underwent surgical intervention (n = 176). All plasma aliquots as well as the standard curve (0–1000 pg/mL) were assayed in duplicate.

### Study objectives and outcomes

The objective of this study was to test the hypothesis that *FXR-1G* > *T* is a predictor of disease severity in CD. The primary endpoints were the risk of surgery and time to surgery in the *FXR-1GG* versus *-1GT* CD populations. Secondary outcomes included other indicators of severity such as number of hospitalizations and number of failed medications, all standardized to per-year of CD diagnosis. Other endpoints included potential differences in plasma concentration of FGF19 in these two genotypic populations. Moreover, we evaluated the rate of surgery, time to surgery, hospitalizations and number of failed medications amongst wild type and variant carriers of the *MDR1 3435C* > *T* (rs1045642) and *PXR -25385C* > *T* (rs3814055).

### Cell-based studies

#### Plasmid constructs

Human (h) FXR-pEF6, hFXR-1GT-pEF6, pRNL-CMV and BSEP-pGL3 were generously provided by Dr. Rommel G. Tirona. Plasmids containing the coding sequence of the estrogen receptor α and β (ERα-pCMV6-XL4 and ERβ-pCMV6-XL4) were purchased from Origene Technologies (Rockville, MD, USA) and subcloned into the pEF6 expression vector using a PCR cloning method^23^. Specifically, the ERα and ERβ open reading frames were respectively amplified by PCR (primers described in Supplementary Table 1), subcloned into pEF6, and their presence confirmed by Sanger sequencing.

#### Transient transfection

Human hepatocarcinoma (HepG2) cells, obtained from American Type Culture Collection (Manassas, VA), were grown in Dulbecco's Modified Eagle Medium (VWR, Radnor, Pennsylvania) supplemented with 10% fetal bovine serum, 2 mM l-glutamine, 100 IU/mL penicillin and 100 µg/mL streptomycin. Cells were seeded in 12-well plates to 90% confluency in 2 days, and subsequently transfected using lipofectin (3 μL/μg of DNA, Lipofectamine 3000, Invitrogen, Carlsbad CA) with one of the following: 250 ng of hFXR-pEF6, ERα-pEF6, ERβ-pEF6, and BSEP-pGL3; or hFXR*-1GT*-pEF6, Erα-pEF6, Erβ-pEF6, and BSEP-pGL3; or blank expression vector.

#### Gene expression

To test the integrity of the subcloned ERα and ERβ after transfection, gene expression was determined after treatment with estradiol (E2) in HepG2 cells. Specifically, cells were transiently transfected using 1000 ng ERα-pEF6, ERβ-pEF6, or empty vector. Prior to RNA extraction, cells were treated for 24 h with 10 nM E2, 100 nM E2, 1 μM E2, or 0.1% DMSO control. Total RNA was extracted with Trizol reagent as per the manufacturer's instructions (Invitrogen). After RNA quantification and confirmation of integrity (DeNovix spectrophotometer) complementary DNA (cDNA) was synthesized using MultiScribe Reverse Transcriptase (RT) and random priming (Applied Biosystems, Foster City, CA). Expression of ERα and ERβ as well as p53 expression was measured by quantitative real time RTPCR (qPCR) using SYBR green detection (FroggaBio, Toronto, ON, Canada) with the ABI 7500 sequence detection system (Applied Biosystems, Foster City, CA). Transcript levels were normalized to 18s rRNA (TaqMan assay ID Hs99999901_s1, Applied Biosystems), and relative expression determined using the 2^−ΔΔCT^ method^24^. Data are presented as fold-change. Primer sequences are detailed in Supplementary Table 1.

#### Luciferase activation assay

HepG2 cells were transfected with ER as described above with the addition of 10 ng of pRNL-CMV per well. After 16 h, cells were washed with Optimem (Gibco, Gaithersburg, MD) and incubated in one of the following: 0.1% DMSO; 10 nM chenodeoxycholic acid (CDCA); 10 μM CDCA and 10 nM β-estradiol (E2); 10 μM CDCA and 100 nM E2; 10 μM CDCA and 1 μM E2. Cell viability, in presence of increasing concentrations of E2 for 24 h (10 nM, 100 nM, 1 μM), was assessed by a luminescent assay (CellTitre-Glo, Promega, Madison WI) prior to the above incubations. Luciferase activities were measured using the Dual Luciferase Reporter Assay Kit (Promega, Madison WI) as per the manufacturer's instructions, and luminescent signal determined (Glomax 20/20 Luminometer, Promega, Madison Wisconsin, USA). Data were normalized by dividing the firefly luciferase activity by the Renilla luciferase activity (F/R ratio). Data are shown as the fold-change in luciferase activity by dividing the F/R ratio by the control.

### Statistical analysis

Given a reported frequency of 8% of the *FXR -1G* > *T* SNV amongst all ethnicities (ExAC database, 1000Genomes database), a minimum of 21 subjects in the *FXR-1G* > *T* variant group (GT) and 347 in the *FXR-1G* > *T* wild type group (GG) would be needed to detect a 30% difference in the incidence of surgery to achieve a power of 80% with a 2-sided *p* value threshold of 0.05.

Statistical analysis was performed using GraphPad Prism version 5, R version 3.5.3, and SPSS version 17.0 statistical software. Allele frequency distribution for the *FXR -1G* > *T*, *MDR1 3435C* > *T* and *PXR -25,385* genotypes were tested for Hardy–Weinberg equilibrium using a χ2 goodness-of-fit test. A Cox proportional-hazards regression model with adjusting covariates was used to assess the influence of *FXR-1T, MDR1 3435T* and *PXR -25385T* variant carrier status on the time to first surgical resection for the total population, males and females and hazard ratios (HR) were expressed with 95% confidence intervals (CI). Risk of surgery associated with *FXR-1GT*, *MDR1 3435T* and *PXR -25385T* variant carrier status was evaluated using a logistic model with adjustment and are expressed as odds ratios (OR) with 95%CI. Covariates that were considered included the following: age, weight, exposure to combined therapy with an immunosuppressant and biologic, any biologic, methotrexate, glucocorticoid or thiopurine exposure, biologic failure^25^ (defined as development of an ADR requiring cessation of the biologic; primary non-response: a lack of improvement in clinical symptoms with induction therapy as defined by their treating physician; or loss of response: a recurrence in disease activity during maintenance therapy despite an adequate response to induction dosing as defined by the treating physician), any drug failure, hospitalizations, smoking history, duration of disease, and pre-operative biologic exposure. A final model was constructed for the total population as well as stratified by sex, adjusting for *FXR-1G* > *T* genotype, age, weight and other, aforementioned covariates.

To test the hypothesis of an association between the *FXR-1G* > *T* SNV and a decrease in the downstream FXR target, FGF19, a Welch's t-test was used to compare FGF-19 plasma concentrations between genotype groups (*FXR -1G* > *T* variant carriers vs wild-type) within the surgical cohort (n = 176). A *p* value < 0.05 was considered significant. A multiple linear regression analysis was used to further evaluate the relationship between *FXR-1T* carrier status, other covariates and the inter-individual variation in FGF-19 plasma concentrations (natural log-transformed) in participants who underwent a surgical intervention. The analysis was performed for the total population as well as stratified by sex. Other covariates assessed included the following: age, weight, disease activity, disease location and history of small bowel resection.

All in vitro experiments were carried out in duplicate and repeated 3 times. Statistical differences between groups were evaluated using a Student's *t* test. A one-way ANOVA with a post hoc Tukey's Multiple Comparison Test was used to compare luciferase activities within groups. A *p* value < 0.05 was considered significant.

### Ethical considerations

The study protocol was approved by the Western University Health Sciences Research Ethics Board. All study subjects provided written informed consent. All methods were performed in accordance with the relevant guidelines and regulations of the Tri-Council Policy Statement.

## Results

### Study population

Subject selection for all participants, as well as baseline characteristics of CD patients, are presented in Supplementary Figure 1 and Table 1. 1,005 patients referred to the London Health Sciences Centre Personalized Medicine Clinic for disease-specific pharmacogenomic testing were screened for inclusion. Individuals without a confirmed diagnosis of IBD or individuals with missing data or those meeting the exclusion criteria were not included in the final analysis (n = 225). Individuals with a diagnosis of UC were excluded from the primary analysis (n = 201); however, they did undergo genotypic analysis to assess *FXR-1G* > *T* carrier status. Their demographic data is summarized in Supplementary Table 2. 542 patients with CD were included in the final analyses of which 176 participants had undergone an intra-abdominal surgical intervention for their CD. The rate of surgery increased with duration of diagnosis (1-year, 10.15%; 5-year, 21.77%; and 10 years after diagnosis, 28.04%). The most common indication for surgery was stricturing disease (*FXR-1GG*, n = 78; *FXR-1GT*, n = 10) followed by fistulizing disease (*FXR-1GG*, n = 45; *FXR-1GT*, n = 5). Surgical interventions included small bowel or ileocolic resections with primary anastomosis and total colectomies with formation of an ileostomy. Supplementary Table 3 summarizes the demographic data for all CD subjects stratified by surgical status and *FXR-1G* > *T* genotype.

**Table 1:** Baseline characteristics.

Characteristics	Subjects with CD (n = 542)
Age, years (mean, range)	41.63 (18–85)
Female sex (%)	321 (59.2)
Weight, kg (mean ± SD)	76.97 ± 19.26
**Disease location**	
Ileal	199 (36.7)
Colonic	99 (18.3)
Ileo-colonic	241 (44.7)
Median HBI (IQR)	4.5 (2–8)
Median disease duration, years (IQR)	4.58 (1.50–13.58)
Smoking history (%)	159 (29.3)
Prescription of a biologic agent (%)	250 (46.1)
Anti-TNF (%)	212 (39.1)
Anti-integrin (%)	20 (3.7)
Anti-IL12/23 (%)	22 (4.1)
Combination therapy (%)	184 (33.9)
Glucorticoid exposure (%)	396 (73.1)
**Immunomodulator exposure**	
MTX (%)	128 (23.6)
Thiopurine (%)	313 (57.7)
Number of drugs failed (mean ± SD)	1.08 ± 1.27
Number of biologics failed (mean ± SD)	0.33 ± 0.72
Surgery (%)	183 (33.8)
Mean number of surgeries (mean ± SD)	0.57 ± 1.09
Hospitalizations (mean ± std)	1.10 ± 2.05
FXR -1GT carrier status (%)	31 (5.7)
FXR-1GG carrier status (%)	511 (94.3)
MDR1 3435CC carrier status (%)	148 (27.3)
MDR1 3435CT/TT carrier status (%)	394 (72.7)
PXR -25385CC carrier status (%)	193 (35.6)
PXR -25385CT/CC carrier status (%)	349 (64.4)

*kg* kilograms, *SD* standard deviation; *CD* Crohn's disease; *HBI* Harvey-Bradshaw Index, *IQR* interquartile range, *TNF* tumor necrosis factor, *IL* interleukin; *MTX* methotrexate, *FXR* farnesoid X receptor, *MDR* multi-drug resistance, *PXR* pregnane X receptor.

**Table 2:** Risk of Surgical Intervention according to *FXR-1G* > *T* genotype.

Risk of event	Cohort (n = 542)		
	Odds ratio	95% CI	*p* value
**Total population**			
Unadjusted	3.34	1.58–7.05	0.002
Adjusted	2.77	1.012–7.58	0.047
**Female**			
Unadjusted	14.87	4.22–52.38	< 0.0001
Adjusted	21.33	3.78–120.3	0.001
**Male**			
Unadjusted	0.49	0.13–1.86	0.29
Adjusted	0.584	0.12–2.82	0.50

Odds ratios were adjusted for the following covariates: age, weight, exposure to combination therapy with a biologic and immunosuppressant agent, any biologic, methotrexate, glucocorticoid or thiopurine exposure, biologic failure, any drug failure, hospitalization, smoking history, duration of disease, and pre-operative biologic exposure.

*CI* confidence interval, *FXR* farnesoid X receptor.

### *FXR-1G* > *T* predicts surgery risk and an earlier progression to surgery in women with CD

Among all subjects in the CD cohort, *FXR -1G* > *T* minor allele (*-1T*) frequency was 2.9%, while allele frequency was in Hardy–Weinberg equilibrium. The incidence of surgery was higher in *FXR-1T* variant carriers compared to wild type individuals (61.3% vs 30.7%). There was a strong association between the presence of the variant T allele and surgical intervention (unadjusted OR = 3.34, 95% CI = 1.58–7.05, *p* = 0.002). After adjustment for baseline covariates and treatment regimens, the *FXR-1GT* genotype remained a significant predictor of surgical risk (Table 2).

**Figure 1:**
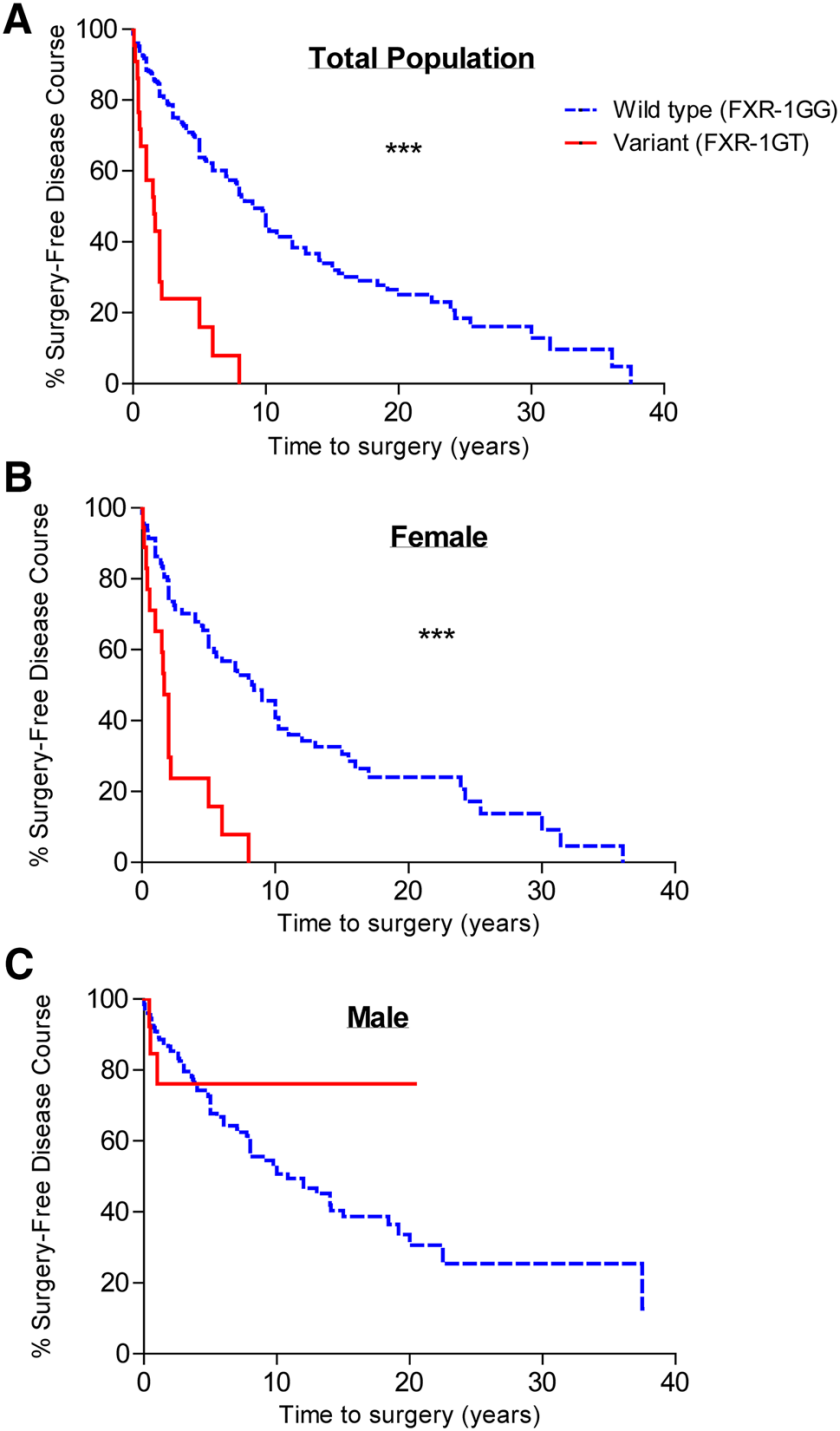
Time to first surgery following the diagnosis of CD stratified by *FXR-1G* > *T* genotype expressed as wild type (GG) or variant (GT) for the total population, n = 542 (**A**); for women, n = 321 (B); and for men, n = 220 (C). ‘***’ represents a *p* value < 0.0001. *CD* Crohn's disease, *FXR* farnesoid X receptor.

*FXR-1T* variant carriers were more likely to go on to a surgical intervention earlier in their disease course compared to wild type individuals (6.08 years ± 7.16 vs 2.69 years ± 2.26; unadjusted HR = 3.00, 95% CI = 1.86–4.83, *p* < 0.0001) (Fig. 1a). Similarly, *FXR-1GT* remained an independent predictor of rapid progression to surgery with adjustment for baseline covariates and drug exposures and responses (Table 3).

**Table 3:** Hazard ratios of surgical risk for *FXR-1GT* versus *FXR-1GG* Genotypes.

Risk of event	Cohort (n = 542)		
	Hazard ratio	95% CI	*p* value
**Total population**			
Unadjusted	3.00	1.86–4.83	< 0.0001
Adjusted	2.67	1.59–4.47	< 0.0001
**Female**			
Unadjusted	6.28	3.62–10.90	< 0.0001
Adjusted	4.33	2.23–8.41	< 0.0001
**Male**			
Unadjusted	0.76	0.24–2.41	0.64
Adjusted	1.37	0.41–4.62	0.61

Hazard ratios were adjusted for the following covariates: age, weight, exposure to combination therapy with a biologic and immunosuppressant agent, any biologic, methotrexate, glucocorticoid or thiopurine exposure, biologic failure, any drug failure, hospitalization, smoking history, duration of disease, and pre-operative biologic exposure.

*CI* confidence interval, *FXR* farnesoid X receptor.

Interestingly, when data were stratified based on sex, the association between variant T allele carrier status and risk of surgical intervention (Table 2) and an earlier progression to surgical intervention (Fig. 1b) was strongest in women, even when adjusted for baseline covariates with an OR of 21.3 (*p* = 0.001, Table 2) and HR of 4.33 (*p* < 0.0001, Table 3). Of the *FXR-1GT* carriers going on to surgery, 84.2% were women compared to only 46.5% in *FXR-1GG* carriers. Conversely in men, *FXR-1G* > *T* genotype was not significantly associated with risk or time to surgery (Fig. 1C, Tables 2, 3).

Other indicators of severity evaluated included the number of drugs related to IBD failed, number of surgeries, and hospitalizations. There was no difference between *FXR-1G* > *T* genotypes in any of these parameters of severity, even when adjusting for weight, age, sex and disease duration (data not shown).

In patients with UC (described in Supplementary Table 2), *FXR-1G* > *T* was not a predictor of surgical risk or risk of early surgery (Supplementary Figure 2). No subjects with a *FXR-1GT* genotype underwent a surgery related to their UC.

### *PXR* and *MDR1* genotypes are not associated with a severe CD phenotype

Additionally, subjects were assessed for the SNVs, *MDR1 3435C* > *T* and *PXR* -*25385C* > *T.* Minor allele frequencies were 53.7% and 40.2%, respectively, in the population. Overall, there was no significant association between either SNV and risk of or time to surgical intervention (Supplementary Figures 3, 4). Furthermore, there was no significant association between either SNV or other indicators of CD severity (number of drugs failed, number of surgeries, hospitalizations).

### *FXR-1G* > *T* is associated with lower FGF19 plasma concentrations in women

**Figure 2:**
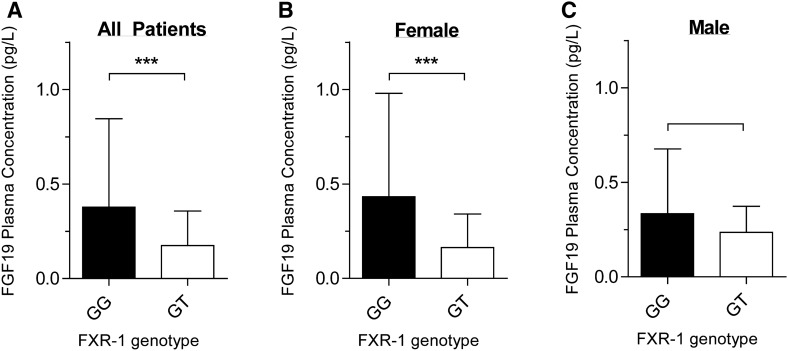
The mean FGF-19 plasma concentrations stratified by *FXR-1G* > *T* genotype, GG or GT for the total population (**A**), for women (**B**) and for men (**C**). Error bars represent the standard deviation. '***' represents a *p* value < 0.0001. Fibroblast growth factor 19, FGF-19; farnesoid X receptor, FXR.

Another objective of this study was to evaluate FGF19 plasma concentrations. Compared to *FXR-1GG* wild type carriers, FGF19 plasma concentrations were lower in *FXR -1GT* variant carriers (Mean ± standard deviation, SD: -1GG carriers = 0.38 ± 0.47 pg/L; -1GT carriers = 0.17 ± 0.18 pg/L, *p* = 0.0008) (Fig. 2a), even after adjustment for the baseline covariates age, sex, weight, disease activity and intestinal resection (*p* < 0.0001, Supplementary Table 4).

Interestingly, when stratified by sex, women with an *FXR-1GT* genotype had a near three-fold lower FGF-19 plasma concentration (GG = 0.43 ± 0.0.54 pg/L; GT = 0.16 ± 0.18 pg/L, *p* = 0.001, Fig. 2b) even with adjustment for the aforementioned covariates (Supplementary Table 5). There was no difference in the male population (Supplementary Table 6).

### ER-FXR interplay

**Figure 3:**
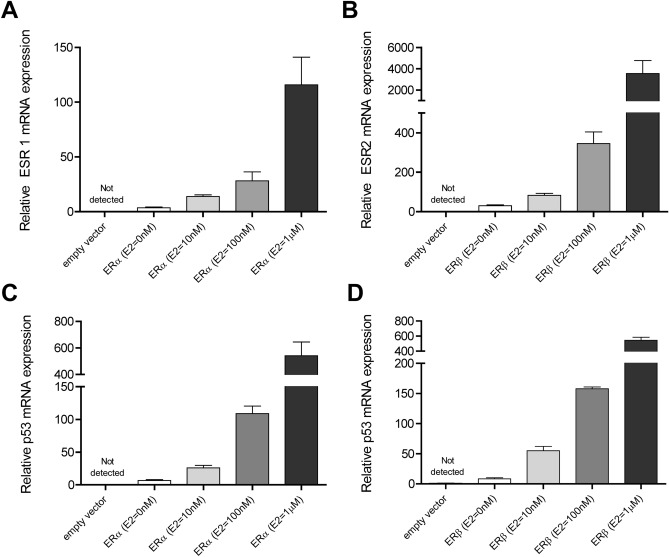
Effect of estradiol on ER-α (ESR1) and ER-β (ESR2) gene expression in addition to the shared ER-target gene p53. HepG2 cells were transiently transfected with ER-α (ESR1), ER-β (ESR2) or vector control (pEF6). ESR1 (**A**), ESR2 (**B**) and p53 (**C**, **D**) mRNA expression was determined by real-time qPCR analysis. *ER* Estrogen receptor, *ESR* estrogen receptor gene, *Hep* hepatoma, *mRNA* messenger ribonucleic acid, *PCR* polymerase chain reaction.

**Figure 4:**
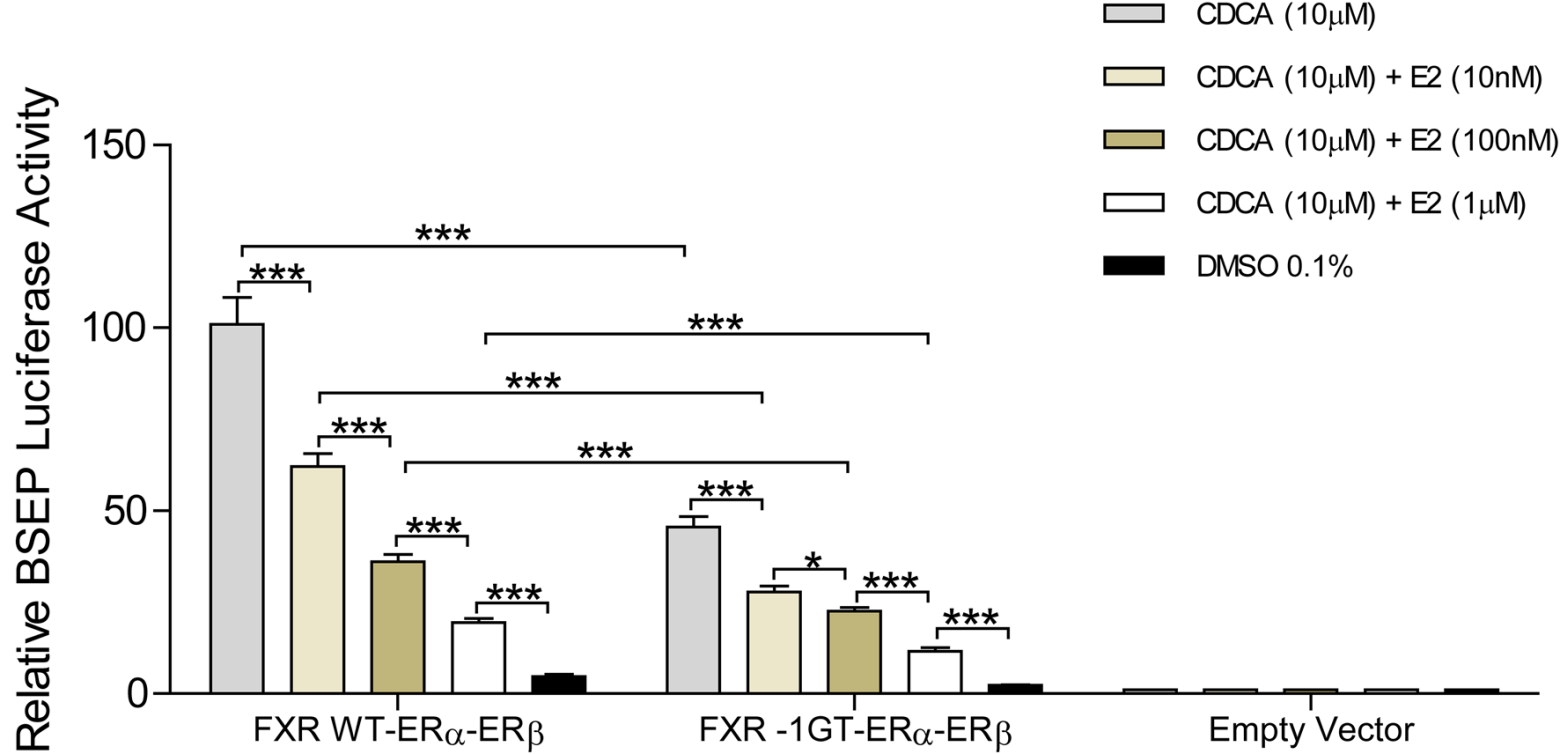
Effect of estradiol on the *FXR*-mediated activation of BSEP-pGL3 basic reporter activity in HepG2 cells co-transfected with ERα-pEF6, ERβ-pEF6 and one of hFXR-pEF6 or hFXR-1GT-pEF6 or a vector control. Cells were incubated with CDCA alone or with CDCA in combination with estradiol in increasing concentrations (10 nM, 100 nM, 1 µM) for 24 h. Data are normalized to renilla luciferase activity and presented as the fold-change relative to the vector control as mean ± SEM. ‘***’ represents *p* values < 0.0001, ‘**’ represents *p* values < 0.001 and ‘*’ represents *p* values < 0.01. Three independent experiments were performed for each model. *CD* Crohn's disease, *FXR* farnesoid X receptor, *BSEP* bile salt export pump, *pGL3* luciferase, *Hep* hepatocarcinoma, *ER* estrogen receptor; *pEF6* expression vector, *h* human, *SEM* standard error of the mean, *CDCA* chenodeoxycholic acid.

To better elucidate a potential molecular basis for the observed effect of *FXR-1G* > *T* genotype on CD prognosis (more frequent and earlier surgery) in female CD patients, we explored a connection between the estrogen receptor-mediated pathway and genetic variation in *FXR*. First, ERα and β expression and function were assessed in our cell-based model after transfection with human ERα and β (Figs. 3, 4). Increased relative mRNA expression of ERα, ERβ and the shared target p53 were observed with increasing concentrations of estradiol (E2) (Fig. 3). To further assess the effect of estrogen on FXR-mediated transcriptional activation of the prototypical FXR target gene *ABCB11* (bile-salt export pump, BSEP)*,* HepG2 cells were co-transfected with human ERα and β, FXR wild type (FXR WT-ERα-ERβ) or variant (FXR-1GT-ERα-ERβ), and a BSEP promoter-luciferase reporter gene. The BSEP-luciferase reporter was robustly activated by FXR WT in the presence of CDCA (10 μM) while for FXR-1GT, reporter luciferase activity was only 45% of wild type *FXR* (Fig. 4, *p* < 0.0001). Moreover, CDCA-induced BSEP-luciferase reporter activity decreased with increasing concentrations of E2 (Fig. 4, *p* < 0.0001), with the greatest decrease seen in variant FXR-expressing cells. No significant differences in cell viability were observed in E2 treated cells (10 nM, 100 nM, 1 μM) compared to control as confirmed by a luminescent assay (CellTitre-Glo, Promega, Madison WI, data not shown).

## Discussion

There is an increasing appreciation of the role of nuclear receptors as determinants of cellular homeostasis as well as in disease pathogenesis, modulation, and therapeutic targets^26^. To date, the clinical relevance of genetic variation in nuclear receptors on disease conditions such as inflammatory bowel disease has not been convincingly demonstrated. This may in part be related to the multifactorial effect of diet, environment, gut microbiome, as well as host genetics to susceptibility of IBD and treatment outcomes.

Two previous studies have evaluated polymorphisms in *FXR* and in the setting of IBD^10,11^. Nijmeijer et al*.* did not find an association between *FXR-1T* variant carrier status and IBD disease presence, location or disease type among 2,355 IBD patients. Similarly, Attinkara et al*.* failed to show an association between *FXR-1T* variant carrier status and IBD susceptibility in a cohort of 1,138 individuals, half of whom had a CD diagnosis. However, in those studies, no assessment was performed to evaluate the link between *FXR-1G* > *T* and disease severity, potential molecular mechanism or sex related differences in disease severity.

In this study, we demonstrate women with CD carrying the *FXR-1GT* genotype are at a much higher risk for progressing on to surgery (adjusted OR = 21.33, 95% CI = 3.78–120.3, *p* = 0.001) compared to men or women with the *FXR-1GG* genotype. Sex-specific differences in CD have only been explored to a limited extent. Most recently, Shah et al. and Severs et al*.* showed sex-specific differences in the incidence of IBD and across several clinical variables associated with disease phenotypes^27–29^. However, there is a paucity of data on how sex differences impact CD diagnosis and disease management^27–29^. This is in contrast to the depth of our understanding of sex-related differences in other chronic diseases such as coronary artery disease, rheumatologic autoimmune diseases, and renal disease^30^. Even in conditions where sex-related differences are known to exist, disparity in disease management is often seen. For example, despite better outcomes in females, women with congestive heart failure receive fewer guideline-based treatments and transplantations compared to men^31^. Similarly, despite a greater risk of stroke, women are less likely to be anticoagulated in the setting of atrial fibrillation compared to men^31^. Such differences and potential disparities have not yet been addressed adequately in CD. There are data to support a slightly higher incidence of CD in women across several populations^32–34^. Oral contraceptive pill (OCP) use has been linked to the onset of CD in a large prospective cohort study of American women as well as to the risk of CD-related surgery in a Swedish registry^35,36^. These findings allude to the importance of a female-specific factor to the susceptibility and severity of CD.

Interestingly, E2, a major female sex hormone and the dominant form of estrogen present during a woman's reproductive years and commonly used in OCP, has been shown to inhibit FXR activity via the ERα in vivo and in vitro^37,38^. However, the molecular mechanism that govern a potential relationship between, for example, OCP use and CD susceptibility, have not been elucidated^35,36^. Interestingly, Goodman et al.^39^ using a murine model of colitis, showed that ER-α loss-of-function resulted in protection from chemically-induced colitis in female mice, directly linking E2-signaling to pathways of IBD. Our findings suggests a plausible molecular mechanism. Our experiments demonstrate that estrogen-mediated ER activation attenuates bile acid-mediated FXR activation, and that the inhibitory effect was more profound in the presence of the variant *FXR*.

We did not observe an association of *MDR1 3435C* > *T* and *PXR* -*25385C* > *T* genotypes with CD severity in our population. To date, there has been a lack of consensus regarding a potential link between CD susceptibility and genetic variation in *MDR1* and *PXR*. Brinar et al*.* and Juyal et al*.* both indicated a link between *MDR1* genetic variation and IBD, while a larger meta-analysis of existing studies refuted the finding^5,12,40^. Similarly, Dring et al.^6^ concluded that *PXR* -*25385C* > *T* was a significant predictor of IBD susceptibility; however, more recent and larger genomic association studies have failed to confirm these original findings^7,9^. Our study confirms a lack of association between functional polymorphisms in either of these genes and CD severity.

Interestingly, the *FXR-1GT* genotype was not associated with risk of surgery or earlier progression to surgery in a cohort of individuals with UC. This may be related to a relatively small cohort size (201) in addition to the low incidence of surgery in this group (18/201). Mechanistically, the study of differences in FXR activation in IBD has commonly identified differences in CD and not UC^11,15,41^. Conversely, one might expect the association between *FXR-1GT* and surgical risk to extend to a UC population given there are many shared inflammatory pathways in CD and UC that are regulated by FXR and abnormalities in epithelial barrier function are documented in both conditions. Further study in a larger cohort may be warranted and other endpoints related to barrier function (disease flares) should be considered.

Importantly, we demonstrate differences in FGF19 plasma concentration, a *FXR* downstream product, between carriers of the *FXR-1GT* genotype versus *-1GG* genotype (Fig. 2). This difference was most pronounced in women, with a near three-fold higher FGF19 plasma concentration in carriers of the wildtype allele compared to carriers of the variant *FXR-1T* allele. We have previously shown in vitro and in vivo that the *FXR-1G* > *T* SNV is associated with reduced activation of downstream products^18^. Similarly, data by Van Mil et al.^20^ demonstrated a concomitant decrease in FXR protein and several of its down-stream gene targets.

A limitation of our study may include that FGF19 quantification was carried out in individuals with small bowel disease who underwent a small bowel resection of varying, non-quantifiable lengths. This may have impacted FGF19 plasma concentrations and thus confounded our findings. Moreover, this is a largely Caucasian study, and as such may not be generalizable to other populations. Furthermore, it is a retrospective and uncontrolled cohort study; thus, it may be affected by selection bias and unknown confounding factors. Lastly, there is no replication cohort for the clinical data set. Additional studies are needed to further assess the relationship between *FXR-1G* > *T* and CD severity.

## Conclusions

This study outlines important new findings of particular relevance to women with CD. Screening female CD patients for *FXR-1T* variant carrier status may be useful for identifying female patients at risk for poor outcomes. Female carriers of the variant allele may benefit from earlier, more aggressive medical management.

## Supplementary information

Supplementary information.
