# Supplementary material for: Genetic variation in the farnesoid X-receptor predicts Crohn’s disease severity in female patients
Source: Sci Rep. 2020 Jul 16;10:11725. doi: 10.1038/s41598-020-68686-9 (PMC7366697; doi:10.1038/s41598-020-68686-9)

Supplementary Material

Genetic variation in the Farnesoid X-Receptor Predicts Crohn's Disease Severity in Female Patients.

Aze Wilson, Qian Wang, Ahmed A. Almousa, Laura E. Jansen, Yun-hee Choi, Ute I. Schwarz, Richard B. Kim

Supplementary Table 1 - 6

Supplementary Figures 1 - 4

SUPPLEMENTARY TABLES:

Supplementary Table 1. Primers for RT-PCR Analysis

**Primer Sets**	**Forward Primer Sequence (5' to 3')**	**Reverse Primer Sequence (5' to 3')**
ERα (*ESR1*)	ATGGAATCTGCCAAGAAGACT	GCGCTTGTGTTTCAACATTCT
ERβ (*ESR2*)	CGATGCTTTGGTTTGGGTGAT	GCCCTCTTTGCTTTTACTGTC
p53 (*TP53*)	CAGACTGCCTTCCGGGTCACTGCC	TCAGTCTGAGTCAGGCCCTTCTGTC

Estrogen receptor, ER; real-time polymerase chain reaction, RT-PCR

Supplementary Table 2. Demographic Characteristics of Patients with UC

Variables	UC cohort (n =201)
**Age, years (mean, range)**	40.92 (18-91)
**Female sex (%)**	101(50.2)
**Weight, kg (mean ± SD)**	75.47 ± 15.63
**Disease location**	
**Pan-colitis**	121(60.2)
**Left-sided colitis**	70 (34.8)
**Proctitis**	5 (2.5)
**Median disease duration, years (interquartile range)**	3.50 (2.00-10.67)
**Smoking history (%)**	23 (11.4)
**Biologic exposure (%)**	117 (57.9)
**Anti-TNF (%)**	60 (29.7)
**Anti-integrin (%)**	19 (9.4)
**Anti-IL12/23 (%)**	0 (0)
**Combination therapy (%)**	31(15.3)
**Glucorticoid exposure (%)**	156 (77.2)
**Immunomodulator exposure**	
**MTX (%)**	15 (7.4)
**Thiopurine (%)**	132 (57.9)
**5-Aminosalicylate exposure (%)**	149 (73.8)
**Number of drugs failed (mean ± SD)**	1.35 ± 1.34
**Number of biologics failed (mean ± SD)**	0.30 ± 0.72
**Surgery (%)**	18 (8.9)
**Mean number of surgeries (mean ± SD)**	0.12 ± 0.40
**Hospitalizations (mean ± SD)**	0.65 ± 0.91
**FXR -1GT carrier status (%)**	12 (5.9)
**FXR -1GG carrier status (%)**	190 (94.1)

Ulcerative colitis, UC; standard deviation, SD; tumor necrosis factor α, TNF; interleukin, IL; methotrexate, MTX; farnesoid X receptor, FXR

Supplementary Table 3. Demographic Characteristics of CD Patients by *FXR -1G>T* Genotype and Surgical Status

Characteristics	Surgical Intervention		No Surgical Intervention	
	**FXR -1GG n = 157**	**FXR-1GT n = 19**	**FXR -1GG n =354**	**FXR-1GT n = 12**
**Age, years (mean, range)**	44.32 (18-85)	41.05(20-70)	40.50 (18-80)	(18-60)
**Female sex (%)**	73 (46.5)	16 (84.2)	229 (64.6)	3 (25.0)
**Weight, kg (mean ± SD)**	76.49 ± 17.35	71.19 ± 18.49	77.31 ± 20.11	80.24 ± 16.70
**Disease location**				
**Ileal**	49 (31.0)	3 (15.8)	145 (40.9)	4 (33.3)
**Colonic**	21 (13.0)	2 (10.5)	76 (21.5)	2 (16.7)
**Ileo-colonic**	87 (56.6)	14 (73.7)	133 (37.6)	6 (50.0)
**Median HBI (IQR)**	6 (3-9)	6 (3-10)	5 (2-8)	8 (4.5-10.5)
**Median disease duration, years (IQR)**	14.00 (6.23-25.00)	12.00(6.50-18.38)	2.25 (1.13-7.75)	2.17 (1.31-5.50)
**Smoking history (%)**	52 (31.1)	1 (5.3)	81 (22.9)	5 (41.7)
**Biologic exposure (%)**	87 (56.1)	13 (68.4)	142 (40.1)	6 (50.0)
**Anti-TNF (%)**	71 (55.4)	9 (47.4)	133 (37.6)	6 (50.0)
**Anti-integrin (%)**	9 (5.7)	1 (5.3)	10 (2.8)	0 (0)
**Anti-IL12/23 (%)**	10 (6.4)	4 (21.1)	7 (2.0)	0 (0)
**Pre-operative biologic exposure (%)**	37 (23.6)	8 (42.1)	-	-
**Combination therapy (%)**	57 (36.3)	6 (31.2)	45 (12.7)	2 (16.7)
**Glucorticoid exposure (%)**	140(89.1)	18 (94.7)	276 (80.0)	9 (75.0)
**Immunomodulator exposure**				
**MTX (%)**	47 (29.9)	7 (36.8)	66 (18.6)	3 (25.0)
**Thiopurine (%)**	98 (62.4)	13 (68.4)	194 (54.8)	5 (41.7)
**Number of drugs failed (mean ± SD)**	1.57 ± 1.54	1.68 ± 1.34	0.89 ± 1.11	0.90 ± 0.99
**Number of biologics failed (mean ± SD)**	0.71 ± 0.99	0.79 ± 1.13	0.15 ± 0.53	0.11 ± 0.33
**Number of surgeries (mean ± SD)**	1.71 ± 1.35	1.68 ± 0.95	-	-
**Time to first surgical intervention, years (mean ± SD)**	6.08 ± 7.16	2.69 ± 2.26	-	-
**Hospitalizations (mean ± SD)**	2.41 ± 2.82	2.21 ± 1.66	0.49 ± 1.41	0.08 ± 0.29

Farnesoid X receptor, FXR; kilograms, kg; standard deviation, SD; Harvey-Bradshaw Index, HBI; interquartile range, IQR; Crohn's disease, CD; tumor necrosis factor, TNF; interleukin, IL; methotrexate, MTX

Supplementary Table 4. Multiple linear regression model for the effect on Ln-transformed FGF-19 plasma concentration for all subjects who underwent a surgery (n = 176, adjusted R^2=0.069)

Variable	β-coefficients	Standard error	P-value
**Intercept**	-2.056	0.797	0.11
**FXR -1T carrier status**	-1.601	0.394	**<0.0001**
**Age**	-0.005	0.008	0.52
**Sex**	0.255	0.252	0.31
**Weight**	0.008	0.007	0.24
**Small bowel resection**	-0.277	0.337	0.41
**Small bowel disease**	0.362	0.402	0.37
**Disease activity**	-0.087	0.248	0.73

Supplementary Table 5. Multiple linear regression model for the effect on Ln-transformed FGF-19 plasma concentration for all female subjects who underwent a surgery (n = 321, adjusted R^2=0.104)

Variable	β-coefficients	Standard error	P-value
**Intercept**	-2.284	1.181	0.06
**FXR -1T carrier status**	-1.897	0.515	**<0.0001**
**Age**	-0.004	0.013	0.76
**Weight**	0.006	0.011	0.59
**Small bowel resection**	-0.137	0.614	0.82
**Small bowel disease**	0.815	0.702	0.25
**Disease Activity**	0.167	0.399	0.68

Supplementary Table 6. Multiple linear regression model for the effect on Ln-transformed FGF-19 plasma concentration for all male subjects who underwent a surgery (n =221, adjusted R^2=0.044)

Variable	β-coefficients	Standard error	P-value
**Intercept**	-0.805	0.968	0.41
**FXR -1T carrier status**	-0.150	0.690	0.89
**Age**	-0.008	0.009	0.37
**Weight**	0.006	0.008	0.49
**Small bowel resection**	-0.527	0.346	0.13
**Small bowel disease**	-0.328	0.420	0.44
**Disease Activity**	-0.444	0.272	0.11

SUPPLEMENTARY FIGURES:

Supplementary Figure 1 Participant flow diagram of the study. Inflammatory bowel disease, IBD; Crohn's disease, CD; ulcerative colitis, UC; number, n; farnesoid X receptor, FXR; multi-drug resistance-1, MDR1; pregnane X receptor, PXR; fibroblast growth factor-19, FGF19.

Supplementary Figure 2 Time to first surgery following the diagnosis of UC (A) and risk of surgery (B) stratified by *FXR-1G>T* genotype expressed as wild type (GG) or variant (GT) in cohort of 201 subjects with UC. Farnesoid X receptor, FXR; ulcerative colitis, UC.

Supplementary Figure 3 Genotype frequency for *MDR1 3435C>T* (Panel 1) and *PXR25385C>T* (Panel 2) separated by the presence or absence of an intra-abdominal surgical intervention related to the CD diagnosis for all subjects (A, D), female subjects (B, E), and male subjects (C, F). Genotypes are expressed as a percentage of the total population of study subjects undergoing surgery (n=176) or not undergoing surgery (n=366). Multi-drug resistance protein 1, MDR1; pregnane X receptor, PXR.

Supplementary Figure 4 Time to first surgery following the diagnosis of CD stratified by *MDR1 3435C>T* (panel 1) and *PXR25385C>T* (panel 2) genotype expressed as wildtype (CC) or variant (CT or TT) for all subjects (A, D), female subjects (B, E), and male subjects (C, F). Multi-drug resistance protein 1, MDR1; pregnane X receptor, PXR; wild type (WT).

Supplementary Figure 1

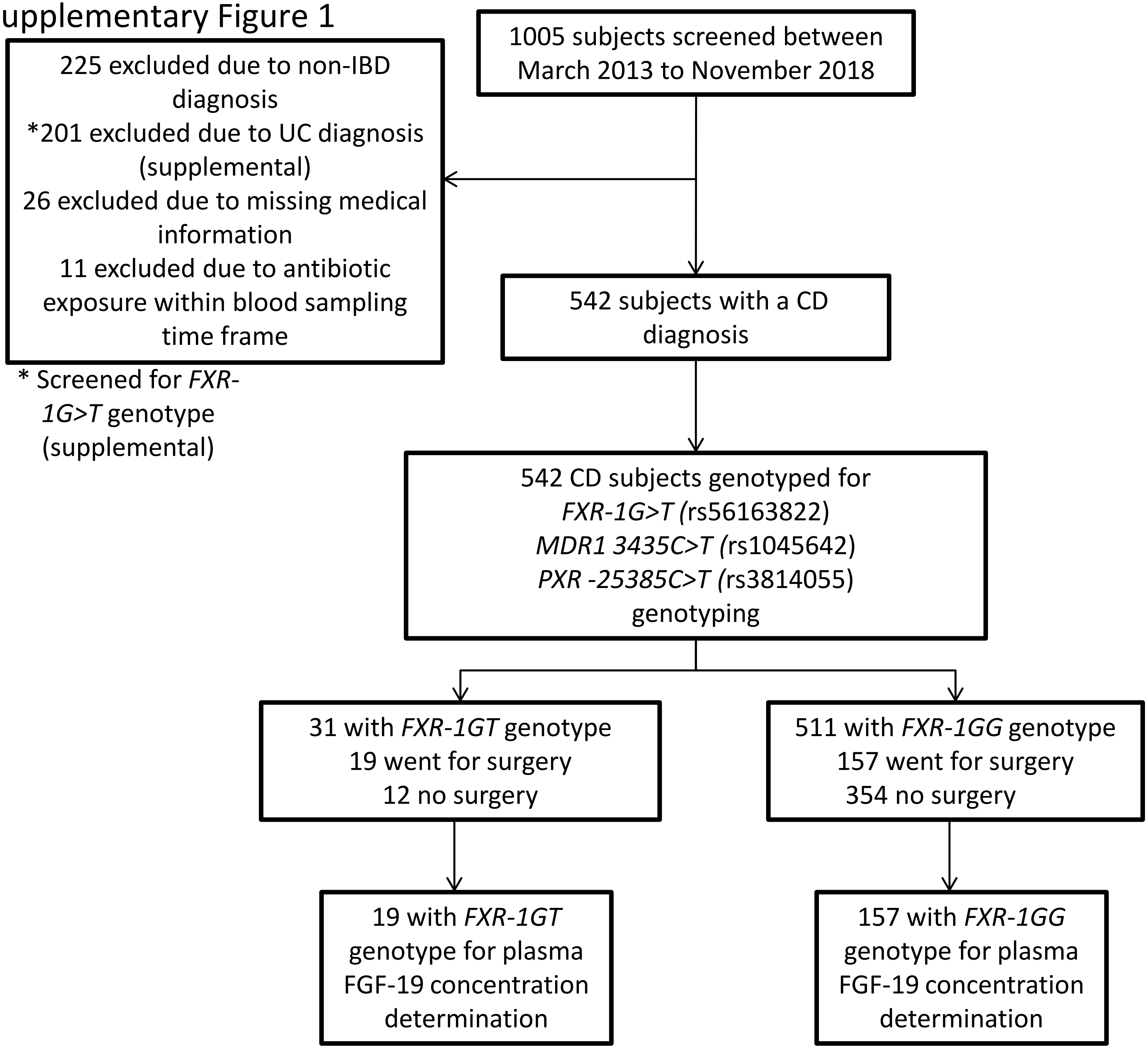

Supplementary Figure 2

A

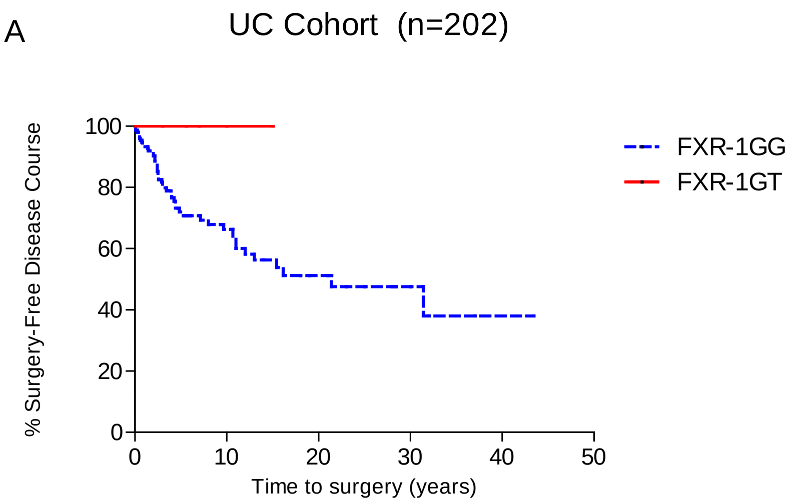

B

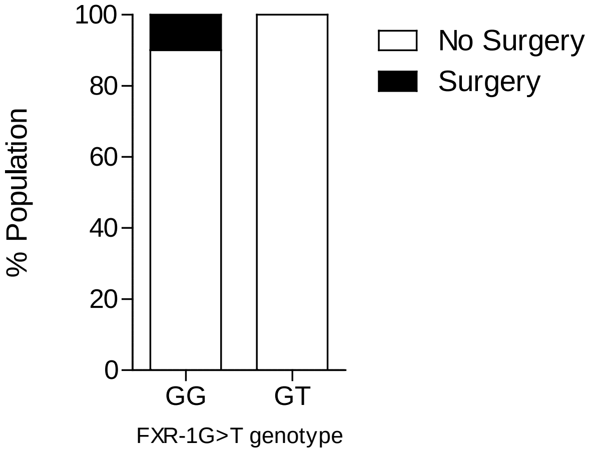

Supplementary Figure 3

1. MDR1 3435C>T

2. PXR 25385C>T

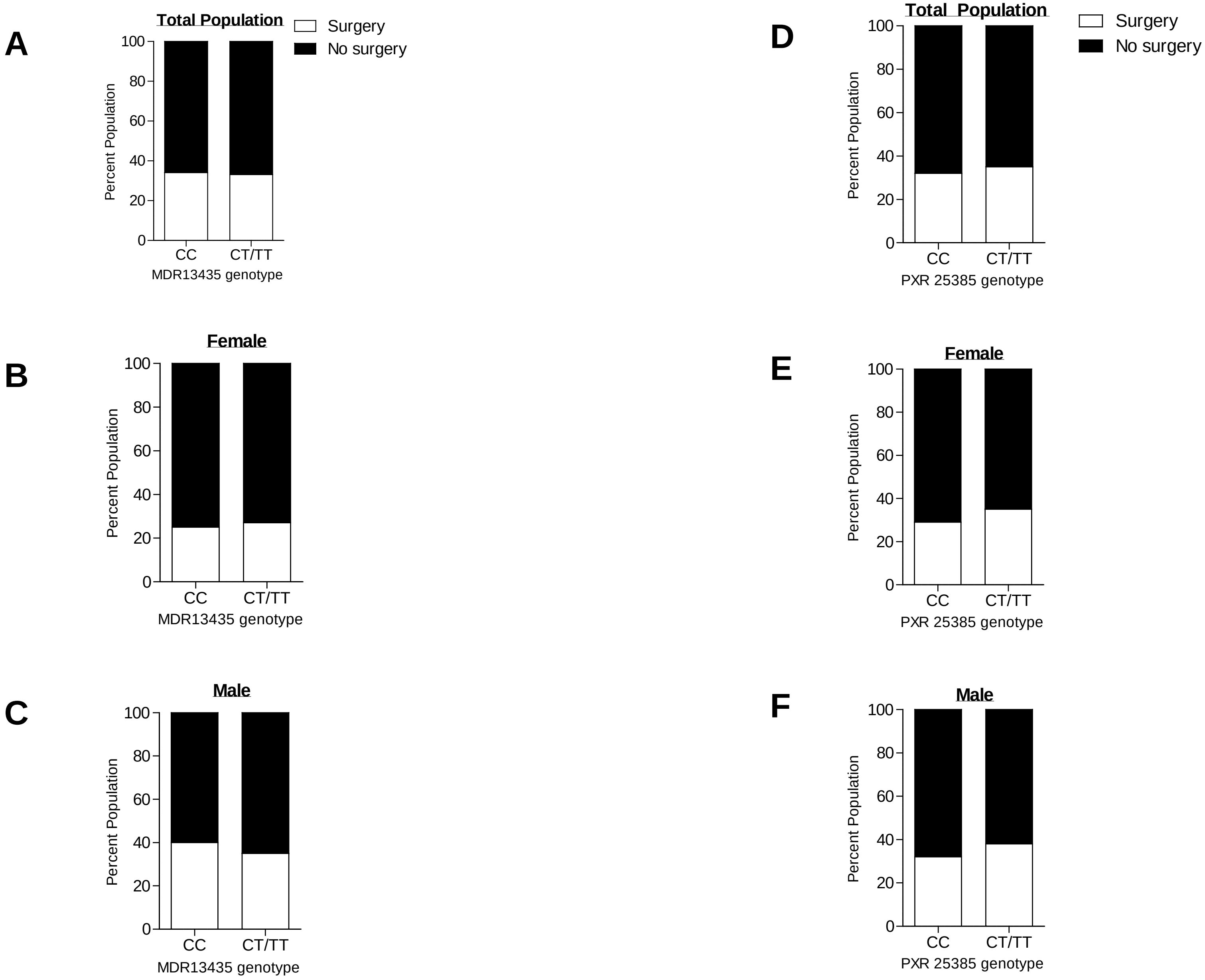

Supplementary Figure 4

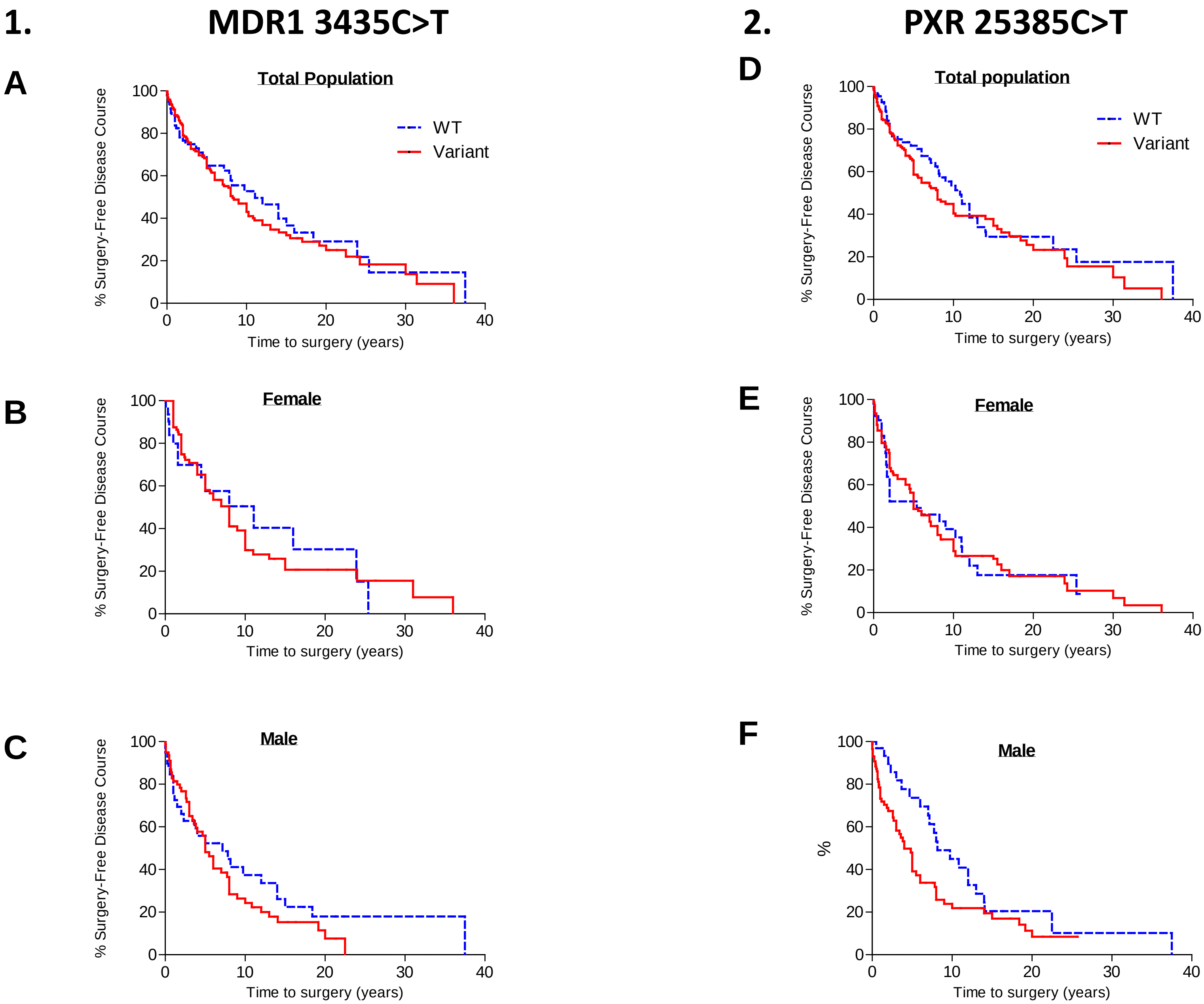

Supplement: Supplementary file 1 — Supplementary information. [file 41598_2020_68686_MOESM1_ESM.pdf]
